# Correction to “The link between Facebook addiction and depression among university students: Evidence from a lower‐middle income country”

**DOI:** 10.1002/hsr2.1790

**Published:** 2024-01-04

1

Sarker MS, Imtiaz A, Haque S, Mahmud KT. The link between Facebook addiction and depression among university students: evidence from a lower‐middle income country. *Health Sci Rep*. 2023;6:e1755. doi:10.1002/hsr2.1755

The affiliations of Shejuti Haque and Kazi Tanvir Mahmud were published incorrectly. The correct affiliations should appear as follows “Department of Economics, Southeast University, Dhaka, Bangladesh.”

We apologize for this error.

